# Isolation and Characterization of Anti-Inflammatory Compounds from *Ficus microcarpa* L.f. Stem Bark

**DOI:** 10.3390/plants12183248

**Published:** 2023-09-13

**Authors:** Mohan Kalaskar, Vivek Redasani, Muniappan Ayyanar, Mahavir Ghante, Sandip Firke, Kapil Agrawal, Vilas Ghawate, Sanjay Surana, Saud Alarifi, Rupesh Chikhale, Shailendra Gurav

**Affiliations:** 1 Department of Pharmacognosy, R. C. Patel Institute of Pharmaceutical Education and Research, Shirpur 425 405, MS, India; kalaskar.mohan@gmail.com (M.K.);; 2 Department of Pharmaceutical Chemistry, YSPM’s Yashoda Technical Campus, Faculty of Pharmacy, Satara 415 001, MS, India; vivek.redasani@gmail.com; 3 Department of Botany, A. V. V. M. Sri Pushpam College, Bharathidasan University, Poondi, Thanjavur 613 503, TN, India; asmayyanar@yahoo.com; 4 Department of Pharmaceutical Chemistry, Guru Ghasidas Vishwavidyalaya, Bilaspur 495 001, CG, India; mhghante@gmail.com; 5 Department of Pharmacognosy, MES College of Pharmacy, Sonai 414 105, MS, India; 6 Department of Zoology, College of Science, King Saud University, P.O. Box 2455, Riyadh 11451, Saudi Arabia; 7 UCL School of Pharmacy, 29-39 Brunswick Square, London WC1N 1AX, UK; r.chikhale@ucl.ac.uk; 8 Department of Pharmacognosy, Goa College of Pharmacy, Goa University, Panaji 403 001, GA, India

**Keywords:** anti-inflammatory activity, *Ficus microcarpa*, catechin, anti-denaturation, COX inhibition

## Abstract

The anti-inflammatory effect of the ethyl acetate extract of *F. microcarpa* bark (EAFMB) was investigated in acute and chronic (21 days) inflammation induced in Wistar albino rats. EAFMB (200 mg/kg b.w.) exhibited comparable anti-inflammatory effects to the reference drug, with a reduction of 59.48% at 4 h in acute inflammation and 83.96% on day 21 in chronic inflammation. Bioassay-guided fractionation using DPPH radical scavenging activity led to isolating and identifying three compounds from EAFMB: oleanolic acid, catechin, and p-hydroxycinnamic acid. All these compounds demonstrated the concentration-dependent inhibition of COX enzymes and the protection of egg albumin from heat-induced denaturation. Catechin exhibited the highest COX inhibition (COX-1 and COX-2 IC_50_ = 9.02 and 50.38 μM, respectively) and anti-denaturation effect (IC_50_ = 27.13 μg/mL) compared to oleanolic acid and p-hydroxycinnamic acid. These isolated compounds are likely responsible for the anti-inflammatory activities of *F. microcarpa* bark.

## 1. Introduction

Inflammation is a complex process caused by infections, hazardous chemical agents, and local immunological responses. Inflammation triggers a reaction through tissue injury, resulting in swelling, redness, heat, pain, and loss of tissue function. It induces vascular dilation, leukocyte reinforcement, and the release of inflammatory mediators, resulting in acute inflammation and, if uncontrolled, chronic inflammatory conditions like diabetes, heart disease, arthritis, and cancer [1]. Arthritis has become a global threat, affecting about 1–2% of the world’s population [2]. It strongly impacts the quality of life of an individual with polyarticular, morning stiffness, destructive and inflammatory cartilage degeneration, the destruction of articular and periarticular bone tissue, and deformity, resulting in a loss of function and inability to work [3]. The conventional treatment options for arthritis available in the market are analgesics, non-steroidal anti-inflammatory drugs (NSAIDs), disease-modifying anti-rheumatic drugs (DMARDs), and corticosteroids. However, these drugs are associated with specific adverse effects such as gastrointestinal upset, ulcers, and bleeding and are also associated with a high cost of treatment [4]. Studies have provided evidence that the long-term use of NSAIDs is associated with severe damage to vital organs, such as reduced kidney function [5], increased risk of cardiovascular complications [6], and chronic liver damage [7]. For these reasons, there is a need for anti-inflammatory drugs to have fewer severe side effects when used for chronic inflammatory diseases as well. 

Indeed, it is a well-established fact that inflammation can result in the release of various inflammatory mediators, including interleukin-6 (IL-6) and C-reactive protein (CRP), as well as cytokines like interleukin-1β (IL-1β) and tumor necrosis factor-alpha (TNF-α) in cases of both acute and chronic inflammation. These mediators play a role in contributing to protein denaturation and aggregation, thereby exacerbating the inflammatory condition. Moreover, inflammation is also responsible for inducing oxidative stress, which, in turn, has the potential to trigger protein denaturation [8,9]. Consequently, the identification and isolation of phytochemicals exhibiting antioxidant properties and the capability to inhibit protein denaturation hold promise in effectively managing both acute and chronic inflammatory states.

*Ficus microcarpa* is renowned for its abundance of triterpenoids, phenylpropanoids, flavonoids, and phenolic acids. Kuo and Li [10] isolated diverse compounds from *F. microcarpa* bark, including triterpenoids, fatty alcohols, steroids, coumarin, flavane, 4-hydroxybenzoate, and a carotenoid-like compound. Additionally, chromatographic and spectroscopic studies identified phenolic compounds such as protocatechuic acid, catechol, p-vinyl guaiacol, syringol, p-propyl phenol, vanillin, and syringaldehyde within the bark, with reported antioxidant and antibacterial properties within in vitro studies [11].

*F. microcarpa* boasts an array of pharmacological attributes, including antioxidant, antibacterial, anticancer, antidiabetic, anti-diarrheal, anti-inflammatory, anti-asthmatic, hepatoprotective, and hypolipidemic activities [12,13,14]. Notably, latex contains chitinase, which lends itself to antifungal properties [15]. *F. microcarpa* has exhibited exceptional antitussive and expectorant potential [16]. Traditionally, *F. microcarpa* has been utilized in folk medicine to address various maladies. In Okinawa, Japan, dried leaves, aerial roots, and the bark of *F. microcarpa*, known as “Gazyumaru”, have been employed to manage perspiration, fever, and pain [11]. In China, *F. microcarpa* referred to as “rong shu”, is used to treat flu, malaria, acute enteritis, tonsillitis, bronchitis, and rheumatism [17]. In South Asia, the plant is a traditional remedy for type-2 diabetes [18]. Furthermore, in India, bark is recognized for its efficacy in treating liver ailments [19]. Our previous research has documented a significant abundance of polyphenols and triterpenoids in the ethyl acetate extract of *F. microcarpa* bark (EAFMB) and their demonstrated antioxidant potential [20]. The literature extensively reports that antioxidants exhibit potent anti-inflammatory activity through various mechanisms [8,9].

Considering the study mentioned above and the lack of existing studies in the literature regarding the anti-inflammatory activity of *F. macrocarpa* bark, assessing the anti-inflammatory potential of the ethyl acetate extract of *F. microcarpa* bark is imperative. This assessment should encompass both acute and chronic inflammation. Additionally, there is a need to perform the fractionation of the extract, evaluate the antioxidant activities of the fractions from *F. microcarpa* bark, and subsequently isolate the bioactive constituents responsible for these effects.

## 2. Results

The percentage yield of the defatted ethyl acetate extract was found to be 7.95%. The preliminary phytochemical analysis of the extract revealed the presence of flavonoids, terpenoids, and phenolics. The acute toxicity test for the EAFMB extract in mice was evaluated at a dose of 2000 mg/kg of body weight. The animals were observed for physical signs of toxicity, such as restlessness, dullness, agitation, increased respiration, writhing, and death. The EAFMB was found to be safe at 2000 mg/kg. This implied a moderate safety profile, and the dosages of the extract employed in this study were calculated at 1/20th (100 mg/kg) and 1/10th (200 mg/kg) to represent low and high doses, respectively.

The effect of EAFMB in acute inflammation using Carrageenan-induced paw edema in rats is presented in Table 1. The EAFMB extract demonstrated a significant (*p <* 0.001) anti-inflammatory effect in a dose-dependent manner compared to the negative control. At 200 mg/kg b.w., the EAFMB extract inhibited the edema caused by Carrageenan from the second hour (52.56%; *p <* 0.001) up to the sixth hour (52.34%; *p <* 0.001) in comparison to a lower dose (100 mg/kg) and the control group (1% CMC p.o.) (Table 1). The results were comparable with the standard drug-treated group.

In chronic inflammation induced by the CFA model, the oral daily administration of EAFMB (100 mg/kg and 200 mg/kg) for 21 days exhibited dose-dependent activity in rats. The group treated with a lower dose of EAFMB (100 mg/kg b.w.) demonstrated a significant reduction in paw thickness from day 14 until the end of the experiment. On the other hand, the effects of EAFMB treatment at a higher dose (200 mg/kg) exhibited substantial inhibition starting from the 8th day (52.77%; *p <* 0.001). This suggests that the anti-inflammatory potential of EAFMB, even at the initial stage of secondary response, experienced progressive inhibition throughout the study (83.96%; *p <* 0.001 on the 21st day) (Table 2). The results from the group treated with EAFMB (200 mg/kg) were comparable to the standard group (*p <* 0.001).

The hematological parameters were studied as markers for inflammation in CFA-induced inflammation. The control group showed elevated hematological parameters, indicating successful inflammation induction. These elevated hematological parameters were reversed in the indomethacin and EAFMB-treated groups (*p* < 0.001) compared to the control group. The group treated with EAFMB (200 mg/kg) showed a significant decrease in their WBC count and ESR (*p* < 0.001), while they showed a significant increase in their RBC and Hb count (*p* < 0.001) compared to the control group. The group treated with EAFMB (100 mg/kg) also showed a significant increase in Hb levels (*p* < 0.01) when compared with the control group (Table 3).

**Figure 1:**
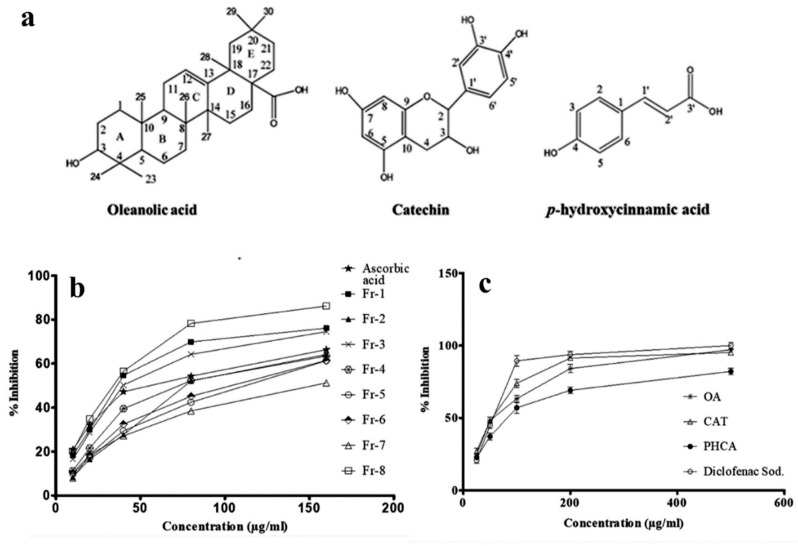
(**a**) Bioactive phytochemicals isolated from EAFMB; (**b**) Free radical scavenging activity of EAFMB fractions; (**c**) In vitro protein denaturation assay of bioactive compounds.

To isolate and identify bioactive chemicals from EAFMB, it was eluted with increasing proportions of hexane: chloroform and chloroform: methanol on a silica gel 60H column (Merck, India, 70–230 mesh, 10 × 50 cm), yielding eight fractions. The DPPH free radical scavenging mechanism is widely accepted for screening the antioxidant activity of plant extracts. In the DPPH assay, the violet-colored DPPH solution was reduced to a yellow-colored product, diphenylpicryl hydrazine, upon the addition of the extract in a concentration-dependent manner. Our results revealed that fractions 1, 3, and 8 of EAFMB exhibited the highest in vitro antioxidant activity with an IC_50_ of 62.78, 71.41, and 49.46 mg/g gallic acid equivalent against the DPPH radical, respectively, when compared to the standard ascorbic acid (Figure 1b). Polyphenols and triterpenoids scavenged DPPH radicals via donating hydrogen. Qualitative chemical tests and TLC of FMC-1 suggest its nature as a triterpenoid.

Similarly, FMC-2 and 3 were revealed to be phenolic compounds. Radical scavenging activities are crucial in preventing the detrimental effects of free radicals in various diseases, including acute and chronic inflammatory conditions. Furthermore, the structural elucidation of isolated compounds was achieved using spectroscopic analysis, identifying them as oleanolic acid, catechin, and p-hydroxycinnamic acid (Figure 1a).

After the purification of bioactive fraction 1, FMC-1 was obtained as a colorless crystalline solid with a melting point of 308–311 °C. FMC-1 exhibited a prominent UV λ max at 202 and 215 nm. The IR spectrum (Figure S1d) exhibited absorption bands for the hydroxyl group at 3414, the carboxylic hydroxyl group at 2926, and a double bond (C=C) at 1693 for the methyl group at 1462 cm^−1^. The ^1^H-NMR spectrum showed seven tertiary methyls cantered at δ 0.77 (1H, s, H3-28), 0.91 (1H, s, H3-29 and H3-30), 0.98 (1H, s, H3-25), 1.05 (1H, s, H3-23), 1.13 (1H, s, H3-26), 1.18 (1H, s, H3-27), and 1.25 (1H, s, H3-24), (all singlets). The carbonylic proton resonated at δ 3.21 (1H, dd, H-3), inferring its α and axial orientation, and a multiplet at δ 5.27 (1H, m, H-12) was indicative of the olefinic proton (Table 4; Figure S1a). ^13^C-NMR spectra revealed the presence of thirty carbon atoms, including eight methyl, ten methylene, five methane, and seven quaternary carbons (Table 4; Figure S1b). The EI-MS spectrum showed a typical fragmenting pattern with a molecular ion peak and base peak at m/z 457 and 248 (100) corresponding to the molecular formula C_30_H_48_O_3_ (Figure S1c).

Bioactive fraction 3 of the successive ethyl acetate extract of *F. microcarpa* yielded FMC-2 as a cream-colored amorphous powder. FMC-2 was confirmed to be tannin using a chemical test, and its melting point was found to be 174–178 °C. Furthermore, it exhibited a characteristic UV λ max at 219 and 276nm. The IR spectrum of FMC-2 indicated the presence of a hydroxyl group with a broad band at 3412 cm^−1^, indicating the presence of a hydroxyl group. Other peaks of the IR spectrum revealed absorptions at 3059 (C-H, aromatic), 1610 (C=C, aromatic), 1514, and 1473 (aromatic ring) cm^−1^ (Figure S2d). The ^1^H NMR spectrum of FMC-3 showed a singlet of the phenolic proton at δ 7.38, a doublet of one proton at 6.92 (1H, d, H-2′), a double doublet of one proton at δ 6.74 (1H, dd, H-6′), doublets of one proton at δ 6.83 (1H, d, H-5′), and one proton integration. The multiplate of the alcoholic hydroxyl proton resonated at δ 3.97 (1H, m, H-3) (Table 4; Figure S2a). The ^13^C-NMR spectrum of FMC-2 revealed the presence of fifteen carbon atoms in the molecules. Spectra made multiplicity assignments, which showed five CH and ten quaternary carbons (Table 4; Figure S2b). The EI-MS showed the molecular ion peak and base peak at m/z 290, 138 (100), along with a specific fragmentation pattern (Figure S2c) corresponding to the molecular formula C_15_H_14_O_6_. 

Purified FMC-3 was obtained as a creamish white powder with a melting point of 188–192 °C. The UV λ max spectrum of FMC-3 showed an absorption band at 310 nm, indicating the presence of an aromatic nucleus in the molecule. The IR spectrum showed the absorption bands at 3358 (Phenolic-OH), 2843 (Acidic-OH), 1666 (Carbonyl-C=O), 1462 cm^−1^ (Aromatic/alkene C=C) (Figure S2d). The ^1^H-NMR spectrum of FMC-3 displayed two resonances in the aromatic region at δ 7.78 (2H, d, H-2, 6), δ 6.91 (2H, d, H-3, 5), with a doublet of one proton at δ 6.31 (1H, d, H-2′; a-position) and δ 7.31 (1H, d, H-1′ b-position) noted (Table 4; Figure S2a). The ^13^C-NMR spectrum of FMC-3 revealed that the downfield signals at δ 169.83 and δ 156.70 were assigned to acid carbonyl and aromatic oxygenated quaternary carbon atoms. By contrast, other signals in the aromatic region at δ 131.06, 118.15, and 128.89 were assigned to aromatic methane and aromatic quaternary carbon atoms. The signals for α-β unsaturated carbon were observed at δ 141.01 and 116.36 (Table 4; Figure S3b). EI-MS of FMC-3 gave the molecular ion peak at m/z 165, corresponding to the molecular formula C_9_H_8_O_3_ (Figure S3c).

The protein denaturation assay revealed that all the isolated phytochemicals were potent inhibitors of thermally induced protein denaturation (Figure 1c). Among the tested phytochemicals, CAT exerted the most substantial anti-denaturation effect (IC_50_ = 27.13 µg/mL), which was equivalent to the reference non-steroidal anti-inflammatory drug diclofenac sodium (IC_50_ = 22.29 µg/mL) followed by OA, and PHCA (Table 5). 

In COX inhibitory assays, all the isolated compounds showed significant inhibition. The COX-1 and 2 inhibitory activities of CAT (IC_50_ = 9.02 and 50.38 μM, respectively) were more significant than the COX-1 and 2 inhibitions shown by other isolated compounds of FMB. CAT exhibited greater COX-2 inhibition compared to standard indomethacin. OA demonstrated a significantly greater COX-1 inhibitory effect than COX-2, while PHCA had a lower IC_50_ for COX-2 inhibition than COX-1 (Table 5).

## 3. Discussion

Several studies have reported the isolation of phytochemicals from *F. microcarpa*. However, despite these claims, no report has been made available on its anti-inflammatory activity and responsible bioactive compounds. Our previous study investigated the antioxidant capacity of four different polarity FMB extracts through a series of antioxidant assays. Strong antioxidant activity was observed in the EAFMB [20]. Thus, by considering the strong correlation between oxidative stress and acute and chronic inflammation, the defatted ethyl acetate extract of *F. microcarpa* bark was evaluated for its anti-inflammatory activity. Carrageenan-induced inflammation is a sensitive, conventional, and accepted animal model for screening newer anti-inflammatory agents. Carrageenan is known for its classic biphasic effect; the first phase is mediated by the release of histamine and serotonin during the first hour and the release of kinins for up to 2.5 h, while the second phase is mediated by the release of prostaglandins from 2.5 to 6 h [21]. In the present study, EAFMB showed the dose-dependent inhibition of the second phase of carrageenan-induced rat paw oedema, which was possibly mediated through the suppression of the cyclooxygenase enzyme, tumor necrosis factor- α, interleukin-1, and interleukin-6 [22]. 

CFA is an inactivated and dried mycobacterium associated with the localized inflammation of the joints, resulting in hypertrophy of the synovial membrane and the progressive and irreversible destruction of the cartilage. CFA-induced inflammation is a biphasic process. The first acute phase (0–10 days) results from the release of various mediators such as histamine, serotonin, kinins, and prostaglandins. These are responsible for oedema and erythema of one or more ankle joints, followed by the involvement of metatarsal and interphalangeal joints. The second chronic phase (11–21 days) is an immunological state characterized by the manifestation of inflammatory mediators such as cytokines (IL-1β and TNF-α), interferon, and platelet-derived growth factors such as swelling and redness. These are responsible for initiating the pain and swelling of the limbs, joints, bone deformation, and joint function disability [23]. EAFMB treatment showed dose-dependent activity in CFA-induced arthritic rats. The group treated with a higher dose (200 mg/kg) showed significant progressive inhibition from the 8th day to the 21st day of the study. This could be related to bioactive phytochemicals that act through the inhibition and release of inflammatory mediators like IL-1β, TNF-α, interferon, and platelet-derived growth factors [24].

In hematological studies of CFA-induced inflammation, there was a rise in the WBC count, erythrocyte sedimentation rate (ESR), and a decrease in RBC and Hb count in the arthritic control group. Changes in hematological parameters in chronic inflammation were mainly caused by drug-induced gastrointestinal blood loss and changes in bone marrow [25]. Furthermore, the decrease in Hb count during chronic inflammation is thought to be caused by lower erythropoietin levels, a reduced response to bone marrow erythropoietin, and the premature destruction of red blood cells. Similarly, an increase in the ESR is linked to the rapid synthesis of endogenous proteins like fibrinogen and/or globulin. An increase in ESR implies an active but unknown disease process [26]. This increase in ESR is caused by stress or inflammation, such as injection, injury, surgery, and tissue necrosis [27]. These elevated hematological parameters have been reversed in indomethacin and EAFMB-treated groups (*p* < 0.001) compared to the control group, which is indicative of the anti-inflammatory potential of EAFMB. 

The characteristic fragmentation pattern of the amyrin skeleton with a double bond at C-12 was shown in the EI-MS spectrum of FMC-1 [28], identifying it as a triterpenoid scaffold. Furthermore, based on the results of physical and spectral data and the available literature, compound FMC-1 was found to be oleanolic acid [29], FMC-2 catechin [30], and FMC-3 p-hydroxycinnamic acid [31].

The anti-inflammatory potential of isolated compounds was evaluated using the in vitro protein denaturation assay and COX inhibition assay. The protein denaturation assay relies on the well-known ability of anti-inflammatory compounds, like NSAIDs, to hinder the thermal coagulation of albumin. Protein denaturation is a well-documented cause of inflammation, and ROSs are recognized stimulants of this process [32]. Oxygen-free radicals play a crucial role in the inflammation process by activating the transcription factor nuclear factor-kB, which triggers the synthesis of inflammatory cytokines and COX-2 [33]. The obtained results demonstrate the significant anti-inflammatory activity of *F. microcarpa* and its phenolic compounds in both COX inhibition and protein denaturation assays. These findings align with studies on other Ficus species, such as *F. racemosa* leaves and bark [34,35,36], *F. religiosa* leaves and bark [37,38], *F. benghalensis* bark [39,40], and *F. carica* [41]. The literature has documented that catechin can exert noteworthy anti-inflammatory properties by modulating the activation or deactivation of inflammation-related oxidative stress signaling pathways. These pathways include tumor necrosis factor-α (TNF-α) induced inflammation, nuclear factor-kappa B (NF-κB), mitogen-activated protein kinases (MAPKs), transcription factor nuclear factor (erythroid-derived 2)-like 2 (Nrf2), and the signal transducer and activator of transcription 1/3 (STAT1/3) pathways [42]. A related study reported that catechin also down-regulates the gene expression of pro-inflammatory cytokines and inflammatory enzymes, leading to the upregulation of anti-inflammatory cytokines [43]. Catechin additionally exerts an antiarthritic effect by mediating cyclic adenosine monophosphate levels, upregulating the expression of Prostaglandin E2 receptor 2, and inhibiting the secretion of proinflammatory cytokines in rats [44]. Moreover, in the present study, the phytochemicals found in *F. macrocarpa*, primarily catechin, synergistically interacted with other polyphenols and triterpenoids, potentially inhibiting oxidative stress, protein denaturation, and COX enzymes. Among the isolated phytochemicals, catechin has emerged as a promising therapeutic agent against various acute and chronic inflammatory conditions and related diseases. The present study validates the traditional claims about this plant and establishes its phytopharmacological relationship.

## 4. Materials and Methods

### 4.1. Plant Collection and Extraction

*F. microcarpa* bark was collected and authenticated from Lonavala, Pune, India (18.7557° N, 73.4091° E). The plant voucher specimen (RCP-42) was deposited at the institutional repository for future reference. *F. microcarpa* bark powder (1.2 kg) was defatted with petroleum ether (60–80°) before extraction with ethyl acetate in a Soxhlet extractor. The ethyl acetate extract of *F. microcarpa bark* (EAFMB) was concentrated to dryness in a rotary evaporator under reduced pressure to obtain a dark reddish-brown polyphenol-rich extract.

### 4.2. Animals

Swiss albino mice (18–25 g) and Wistar albino rats (180 to 200 g) of either sex were used. The animals were housed under standard conditions of temperature (23 ± 1 °C), relative humidity (55 ± 10%), 12 h/12 h light/dark cycles and fed with a standard pellet diet (Amrut, Pranav Agro Industries Ltd., Sangli, India). The experimental animals were fasted for 12 h before the experiment and received water ad libitum. All animal experiments were carried out according to the institutional animal ethical committee (approval letter no. RCPIPER/2018-19/32).

### 4.3. Drugs and Chemicals

DPPH radicals, Carrageenan, and Freund’s complete adjuvant were purchased from Sigma Chemical Company, Bengaluru, India. Diclofenac sodium and indomethacin were obtained as gift samples from Hindustan Ciba-Geigy Ltd., Mumbai, India, and MKR Testing Laboratory, Mumbai, India. All the solvents used for extraction and fractionation were of the AR grade and purchased from Rankem, Avantor, Thane, Mumbai, India. 

### 4.4. Acute Toxicity 

An acute toxicity study of the EAFMB extract was determined in albino mice according to OECD guidelines No. 425 [23]. The animals were fasted overnight. One group was maintained as a control and given 0.5% Tween-80. The test extracts were administered orally at a maximum 2000 mg/kg dose. The animals were observed continuously for 1 h for any gross behavioral changes and death, if any, intermittently for the next 6 h and then again at 24 h after dosing with the test extracts.

### 4.5. Anti-Inflammatory Activities 

#### 4.5.1. Carrageenan-Induced Acute Inflammation in Rats

The acute anti-inflammatory activity of EAFMB was evaluated using the carrageenan-induced rat paw edema model [23]. Experimental animals were randomly divided into four groups, with six animals in each group. Group I (the control group) received a vehicle (1% CMC). Group II (standard group) received indomethacin at 10 mg/kg. Groups III and IV (test group) were given EAFMB at 100 and 200 mg/kg p.o doses [4], respectively. The drugs were administered orally 1 h before the carrageenan injection. The 0.1 mL of freshly prepared carrageenan suspension was injected into the left hind paw of each rat. The paw volume was measured using a Plethysmometer (Ugo Basile 2470, Italy) at 1, 2, 3, 4, 5, and 6 h after carrageenan administration. The results were expressed as a percentage of the increase in paw swelling and a percentage of the inhibition of paw swelling compared to the initial hind paw thickness.

$$\%\;\mathrm{Increase}\;\mathrm{in}\;\mathrm{Paw}\;\mathrm{Volume}\;=\frac{\mathrm{Final}\;\mathrm{Paw}\;\mathrm{Volume}\;\mathrm{at}\;\mathrm{specific}\;\mathrm{time}\;\mathrm{point}\;-\;\mathrm{Intial}\;\mathrm{Paw}\;\mathrm{Volume}\;\mathrm{at}\;0\;h}{\mathrm{Intial}\;\mathrm{Paw}\;\mathrm{Volume}\;\mathrm{at}\;0\;h}\times 100$$

$$\%\;\mathrm{Inhibition}=\frac{\mathrm{Paw}\;\mathrm{Volume}\;\mathrm{at}\;\mathrm{of}\;\mathrm{Control}\;-\;\mathrm{Paw}\;\mathrm{Volume}\;\mathrm{of}\;\mathrm{treated}\;\mathrm{group}}{\mathrm{Intial}\;\mathrm{Paw}\;\mathrm{VoluPaw}\;\mathrm{Volume}\;\mathrm{at}\;\mathrm{of}\;\mathrm{Control}\;\mathrm{me}\;\mathrm{at}\;0\;h}\times 100$$

#### 4.5.2. Complete Freund’s Adjuvant (CFA)-Induced Chronic Inflammation in Rats 

The chronic anti-inflammatory activity of EAFMB was evaluated using the CFA-induced rat paw edema model [23]. Wistar rats of either sex (180 to 200 g) were randomly divided into four groups, each comprising six animals. The control group (Group I) received only a vehicle (0.5% CMC, p.o.). Indomethacin at 10 mg/kg, p.o., was used as a standard drug (Group II). Groups III to IV were treated with EAFMB (100 and 200 mg/kg, p.o., respectively) once daily for 21 days. On day 0, chronic inflammation, i.e., arthritis, was induced via injecting 0.1 mL of CFA containing 0.5% of dry heat-killed *Mycobacterium tuberculosis* in sterile paraffin oil into the sub-plantar region in the left hind paw of rats. The animals were evaluated for the following parameters:

- % of a rise or inhibition of oedema

After the CFA injection, the paw oedema was measured using a plethysmograph on day 0 and thereafter on days 4, 8, 14, and 21. The results were expressed as the percent rise in and percent inhibition of paw oedema as per the formulae mentioned in the acute inflammation study.

- Hematological Parameters

On the 21st day, blood was withdrawn through a retro-orbital plexus puncture from all treated groups under light ether anesthesia. The hematological parameters like hemoglobin content, total WBC count, ESR, and RBC were analyzed using Yumizen H550, HORIBA Hematology Analyzer, France.

### 4.6. Bioactivity-Guided Fractionation and Isolation of Phytochemicals 

**Figure 2:**
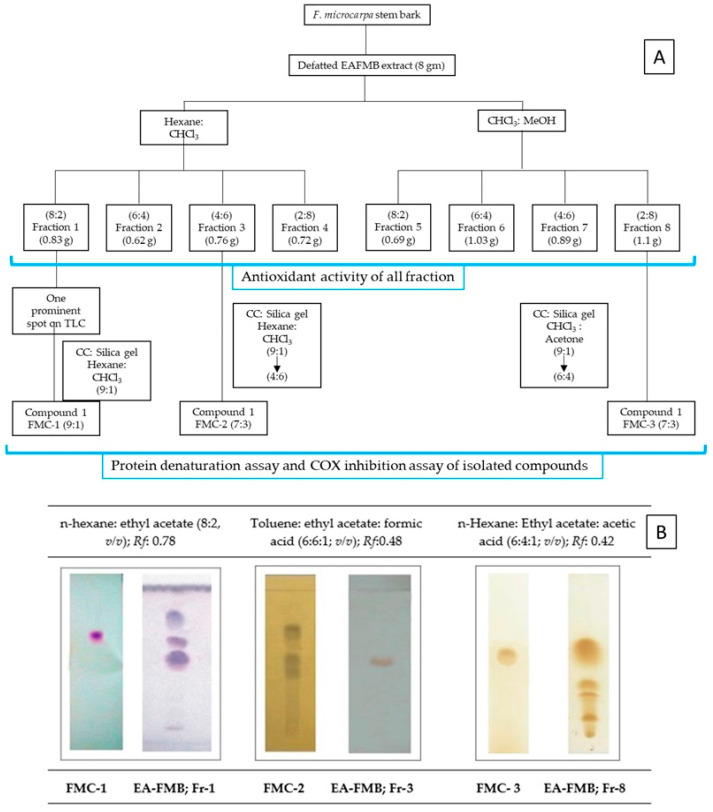
(**A**) Scheme for isolation of bioactive phytochemicals from defatted EAFMB extract; (**B**) Comparative TLC of isolated phytochemicals with fractions.

Eight grams of EAFMB were fractionated using a glass column packed with silica gel 60H (Merck 70–230 mesh, 10 × 50 cm). The elution process involved a combination of hexane/chloroform in ratios of 8:2, 6:4, and 2:8 (800 mL each), followed by chloroform/methanol in ratios of 8:2, 6:4, and 2:8 (800 mL each) to increase polarity (Figure 2). A total of eight fractions were collected and assessed using TLC. Among these, fractions 1, 3, and 8 exhibited the highest antioxidant potential, as determined through the DPPH* radical scavenging assay [45]. This prompted further emphasis on fractionation and purification to isolate bioactive phytochemicals. The most active fractions, namely fraction 1 (820 mg) and fraction 3 (760 mg), were resuspended in 10 mL of chloroform, while fraction 8 (1100 mg) was resuspended in 20 mL of methanol. Subsequently, these fractions underwent additional fractionation using silica gel 60H (Merck 70–230 mesh, 5 × 50 cm). Fraction 1 was eluted with a gradually increasing proportion of hexane to chloroform (10:0 → 8:2) combinations. The fraction that eluted at an 8:2 proportion was monitored using TLC with the solvent system of toluene/ ethyl acetate/ formic acid (6:6:1; *v*/*v*) on silica gel G, revealing a single prominent spot when detected using anisaldehyde sulfuric acid. Further recrystallization using dichloromethane yielded a crystalline compound denoted as FMC-1. Fraction 3 was eluted from the silica gel column with a gradual increase in the proportion of hexane to chloroform (9:1 → 4:6) mixtures. The column fractions were monitored using TLC with the solvent system toluene/ ethyl acetate/ formic acid (6:6:1; *v*/*v*) on silica gel G. The fraction that eluted at a 7:3 solvent mixture showed a single prominent spot identified using the 10% FeCl_3_ reagent. Furthermore, recrystallization using ethanol yielded a crystalline compound to obtain the crystalline compound FMC-2. Similarly, fraction 8 was eluted with chloroform to acetone (9:1 → 6:4) on the silica gel column. The fraction that eluted at a 7:3 mixture of solvents showed a single spot-on TLC with the solvent system n-hexane/ ethyl acetate/ acetic acid (6:4:1; *v*/*v*) on silica gel G, which was detected using 10% ferric chloride reagents and designated as FMC-3. 

#### Identification of Isolated Phytochemicals

The isolated compounds, i.e., FMC-1, 2, and 3, were identified using spectral data from and confirmed by comparing spectroscopic data from FT-IR, UV-Vis, GC-MS, ^1^H-NMR, and ^13^C-NMR spectroscopy with data from previously published studies. The melting point of the isolated compounds was determined using a Digital Melting Point apparatus. A small amount of the samples was placed in the capillary, and the melting point of the samples was recorded. FT-IR spectra of the isolated compounds were recorded on the FT-IR instrument (Shimadzu 8400s, Kyoto Japan). Moisture-free samples were directly placed on the sample holder plate, and the spectra were recorded. ^1^H NMR and ^13^C NMR of the isolated compounds were recorded on a Varian 300 MHz Mercury Plus spectrometer, and chemical shifts were expressed in δ ppm using TMS as an internal standard. An Agilent 7890 instrument was used for GC, and a Joel Accu TOF GCV instrument was used for MS. The inert gas of helium (99.999%) was used as the carrier gas with a 1 mL/min flow rate. An HP5 column with specifications of a length of 30 mm, an internal diameter of 0.32 mm, and a film thickness of 0.25 mm at SAIF, IIT, Bombay, India, was used. The isolated compounds FMC-1, 2, and 3 were identified as oleanolic acid (OA), catechin (CAT), and p-hydroxycinnamic acid (PHCA), respectively. Subsequently, the isolated phytochemicals were evaluated within in vitro protein denaturation and COX-1 and COX-2 inhibition assays.

### 4.7. In-Vitro Anti-Inflammatory Activity Isolated Phytochemicals 

#### 4.7.1. Protein Denaturation Assay

The anti-inflammatory activity of FMC-1, FMC-2, and FMC-3 was evaluated by the protein denaturation method described by Gavit et al. [46]. Diclofenac sodium, a non-steroidal anti-inflammatory drug, was used as a standard drug. The reaction mixture consisting of 2 mL of different concentrations of isolated compounds (100–200 µg/mL) or standard diclofenac sodium (100 and 200 µg/mL) and 2.8 mL of phosphate-buffered saline (pH 6.4) was mixed with 2 mL of egg albumin (from fresh hen’s egg) and incubated at 27 ±1 °C for 15 min. Denaturation was induced by keeping the reaction mixture at 70 °C in a water bath for 10 min. After cooling, the absorbance was measured at 660 nm using double distilled water as a blank. Each experiment was performed in triplicate, and the average was taken. The percentage inhibition of protein denaturation was calculated using the following formula.

$$\%\;Inhibition=\frac{At-Ac}{Ac}\times 100$$

where, At = absorbance of test sample; Ac = absorbance of control.

#### 4.7.2. Cyclooxygenase (COX) Inhibitor Screening Assay

A colorimetric COX (ovine) Inhibitor Screening Assay Kit (Cayman Chemical Co., Cat. No. 760111, USA) was used to evaluate the inhibitory activity against COX-1 and COX-2 [47]. The enzymes were pre-incubated for 5 min at 25 °C with the test compounds (5, 10, 50, 100, and 500 µg/mL) before the addition of arachidonic acid (final concentration 1.1 mM) and N,N,N′,N′-tetramethyl-p-phenylenediamine, (TMPD) followed by incubation for 5 min at 25 °C. The test compounds’ COX-1 or COX-2 inhibitory activities were measured by monitoring the production of TMPD when oxidized at 590 nm. The IC_50_ is the concentration of tested compounds calculated using the regression equation.

## 5. Conclusions

*F. microcarpa* is a well-known bioactive plant in traditional medicinal plants. The present investigation aimed to determine the anti-inflammatory and anti-arthritic activity of the *F. microcarpa* bark extract. This study confirmed that *F. microcarpa* bark possessed significant anti-inflammatory and anti-arthritic activity. The bioassay-guided isolation of the ethyl acetate extract of *F. microcarpa* bark yielded oleanolic acid, catechin, and p-hydroxycinnamic acid. Thus, the current study’s results suggest that oleanolic acid, catechin, and p-hydroxycinnamic acid from *F. microcarpa* could be responsible for their anti-inflammatory activity in acute and chronic inflammation. However, further studies are required to determine their exact mechanism of action. 

## Figures and Tables

**Table 1:** Effects of EAFMB in carrageenan-induced acute inflammation.

Treatment	% Rise in Paw Oedema (% Inhibition)					
	1 h	2 h	3 h	4 h	5 h	6 h
Control ^a^	16.84 ± 2.39	29.96 ± 1.67	37.56 ± 1.25	44.79 ± 0.65	49.29 ± 0.96	52.22 ± 0.62
Standard ^b^ (10 mg/kg)	14.28 ± 0.42 * (27.78)	15.85 ± 0.41 *** (56.84)	18.19 ±0.36 *** (57.97)	21.01 ± 0.51 *** (59.39)	26.18 ± 0.63 *** (54.37)	31.2 ± 0.81 *** (48.18)
EAFMB (100 mg/kg)	16.05 ± 0.52 *** (19.84)	18.75 ± 1.21 *** (49.57)	24.96 ± 1.27 *** (43.12)	26.52 ± 1.42 *** (58.48)	28.62 ± 1.89 *** (50.82)	29.58 ± 0.75 *** (51.56)
EAFMB (200 mg/kg)	13.87 ± 0.36 *** (26.98)	16.73 ± 0.26 *** (52.56)	18.26 ± 0.66 *** (56.16)	20.67 ± 0.52 *** (59.39)	23.51 ± 0.34 *** (57.38)	27.57 ± 0.37 *** (52.34)

Values are expressed as mean ± SEM, n = 6. Statistical differences were calculated using one-way ANOVA followed by Dunnett’s test. *** *p <* 0.001; * *p <* 0.05; ^a^ Control group animal received vehicle p.o.; ^b^ Standard group treated with indomethacin p.o.

**Table 2:** Effects of EAFMB in CFA-induced chronic inflammation.

Treatment	% Rise in Paw Oedema (% Inhibition)			
	4th Day	8th Day	14th Day	21st Day
Control ^a^	66.11 ± 2.90	81.27 ± 4.10	85.83 ± 5.22	81.11 ± 3.54
Standard ^b^ (10 mg/kg)	41.92 ± 1.46 *** (46.78)	27.95 ± 1.58 *** (70.16)	14.77 ± 1.39 *** (84.48)	11.99 ± 0.89 * (87.35)
EAFMB (100 mg/kg)	53.84 ± 1.18 *** (35.88)	54.57 ± 1.36 *** (46.94)	43.84 ± 1.17 *** (59.27)	30.52 ± 2.62 * (69.84)
EAFMB (200 mg/kg)	47.48 ± 1.92 *** (41.73)	46.81 ± 1.48 *** (52.77)	27.36 ± 2.33 *** (73.93)	15.49 ± 1.40 * (83.96)

Values are expressed as mean ± SEM, n = 6. Statistical differences were calculated using one-way ANOVA followed by Dunnett’s test. *** *p <* 0.001; * *p <* 0.05; ^a^ Control group animal received vehicle p.o.; ^b^ Standard group treated with indomethacin p.o.

**Table 3:** Effect of EAFMB on hematological parameters in CFA-induced chronic inflammation rats.

Treatment	ESR (mm/h)	RBC (millions/cubic mm)	WBC (10^3^/cubic mm)	Hb (g%)
Control ^a^	11.73 ± 0.34	5.63 ± 0.36	14.42 ± 0.35	9.33 ± 0.29
Standard ^b^ (10 mg/kg)	5.42 ± 0.31 ***	9.58 ± 0.27 ***	6.37 ± 0.36 ***	14.62 ± 0.27 ***
EAFMB (100 mg/kg)	7.53 ± 0.37 ***	7.52 ± 0.22 ***	11.93 ± 0.40 ***	11.13 ± 0.36 **
EAFMB (200 mg/kg)	6.18 ± 0.28 ***	9.13 ± 0.36 ***	8.05 ± 0.35 ***	14.07 ± 0.46 ***

Values are expressed as mean ± SEM, n = 6. Statistical differences were calculated using one-way ANOVA followed by Dunnett’s test. *** *p <* 0.001; ** *p <* 0.01; ^a^ Control group animal received vehicle p.o.; ^b^ Standard group treated with indomethacin p.o.

**Table 4:** ^1^H and ^13^C NMR spectral data of the FMC-1, 2, and 3 (oleanolic acid, catechin, and p-hydroxycinnamic acid).

FMC-1			FMC-2			FMC-2		
Carbon Position	^1^H (δ)	^13^C (δ)	Carbon Position	^1^H (δ)	^13^C (δ)	Carbon Position	^1^H (δ)	^13^C (δ)
1		37.01	1			1		128.89
2		27.08	2	4.60 (1H, *d*)	81.39	2	7.78 (2H, *d*)	131.06
3	3.21 (1H, *dd*)	78.95	3	3.97 (2H, *m*)	67.74	3	6.91 (2H, *d*)	118.15
4		38.31	4		27.14	4		
5		55.12	5		155.49	5	6.91 (2H, *d*)	118.15
6		18.21	6	5.82 (1H, *d*)	95.81	6	7.78 (2H, *d*)	131.06
7		32.35	7		156.48	1′		116.36 (β-position)
8		38.68	8	6.01 (1H, *d*)	94.73	2′	6.31 (1H, *d*, H-2; α-position)	141.01 (α-position)
9	1.59 (1H, *t*)	47.55	9		156.15	3′	7.31 (1H, *d*, H-1′ β-position)	169.83 (-COOH)
10		33.71	10		99.47			
11		22.82	1′		130.3			
12	5.27 (1H, *m*)	122.56	2′	6.92 (1H, *d*)	114.07			
13		143.52	3′		144.66			
14		41.50	4′		144.79			
15			5′	6.83 (1H, *d*)	115.11			
16		23.51	6′	6.74 (1H, *dd*)	119.16			
17		45.78						
18	1.86 (1H, *t*)	39.18						
19		46.45						
20		29.65						
21		33.01						
22		30.61						
23	1.05 (1H, s)	28.07						
24		15.47						
25	0.98 (1H, s)	15.25						
26	1.13 (1H, s)	17.04						
27	1.18 (1H, s)	25.88						
28	0.77 (1H, s)	183.54						
29	0.91 (1H, s)	32.51						
30	0.91 (1H, s)	23.32						

**Table 5:** Inhibitory effects of compounds isolated from *F. microcarpa* on ovine COX-1 and COX-2 activity in vitro.

Compounds	Protein Denaturation Assay (IC_50_, µg/mL)	COX Inhibition (IC_50_, μM)	
		COX-1	COX-2
Oleanolic acid	69.56	377.57	85.33
Catechin	27.13	9.02	50.38
*P*-hydroxycinnamic acid	141.28	228.77	245.68
Indomethacin ^a^	22.29	4.65	178.75

^a^ Positive control.

## Data Availability

Not applicable.

## Supplementary Materials

The following supporting information can be downloaded at: https://www.mdpi.com/article/10.3390/plants12183248/s1.
