# Supplementary material for: Isolation and Characterization of Anti-Inflammatory Compounds from Ficus microcarpa L.f. Stem Bark
Source: Plants (Basel). 2023 Sep 13;12(18):3248. doi: 10.3390/plants12183248 (PMC10538222; doi:10.3390/plants12183248)

Supplementary File

Isolation and Characterization of Anti-Inflammatory Compounds from *Ficus microcarpa* L.f. Stem Bark

Mohan Kalaskar [1], Vivek Redasani [2], Muniappan Ayyanar [3], Mahavir Ghante [4], Sandip Firke [1], Kapil Agrawal [1], Vilas Ghawate [5], Sanjay Surana [1], Saud Alarifi [6], Rupesh Chikhale [7] and Shailendra Gurav [8,*]

[1] Department of Pharmacognosy, R. C. Patel Institute of Pharmaceutical Education and Research, Shirpur 425 405, MS, India; kalaskar.mohan@gmail.com (M.K.); sandipdfirke@gmail.com (S.F.); contactkap@gmail.com (K.A.); sjsurana@yahoo.com (S.S.)
[2] Department of Pharmaceutical Chemistry, YSPM's Yashoda Technical Campus, Faculty of Pharmacy, Satara 415 001, MS, India; vivek.redasani@gmail.com
[3] Department of Botany, A. V. V. M. Sri Pushpam College, Bharathidasan University, Poondi, Thanjavur 613 503, TN, India; asmayyanar@yahoo.com
[4] Department of Pharmaceutical Chemistry, Guru Ghasidas Vishwavidyalaya, Bilaspur 495 001, CG, India; mhghante@gmail.com
[5] Department of Pharmacognosy, MES College of Pharmacy, Sonai 414 105, MS, India; ghawatevilas@gmail.com
[6] Department of Zoology, College of Science, King Saud University, P.O. Box 2455, Riyadh 11451, Saudi Arabia; alarifi_saud@yahoo.com
[7] UCL School of Pharmacy, 29–39 Brunswick Square, London WC1N 1AX, UK; r.chikhale@ucl.ac.uk
[8] Department of Pharmacognosy, Goa College of Pharmacy, Goa University, Panaji 403 001, GA, India
* Correspondence: shailendra.gurav@nic.in; Tel.: +91-9970835019

Citation: Kalaskar, M.; Redasani, V.; Ayyanar, M.; Ghante, M.; Firke, S.; Agrawal, K.; Ghawate, V.; Surana, S.; Alarifi, S.; Chikhale, R.; et al. Isolation and Characterization of Anti-Inflammatory Compounds from *Ficus microcarpa* L.f. Stem Bark. *Plants* **2023**, *12*, 3248. https://doi.org/10.3390/plants12183248

Academic Editors: Farid Chemat, Noureddine Allouche and Nathan Téné

Received: 18 August 2023
Revised: 9 September 2023
Accepted: 11 September 2023
Published: 13 September 2023

Copyright: © 2023 by the authors. Submitted for possible open access publication under the terms and conditions of the Creative Commons Attribution (CC BY) license (https://creativecommons.org/licenses/by/4.0/).

Abstract: The anti-inflammatory effect of the ethyl acetate extract of *F. microcarpa* bark (EAFMB) was investigated in acute and chronic (21 days) inflammation induced in Wistar albino rats. EAFMB (200 mg/kg b.w.) exhibited comparable anti-inflammatory effects to the reference drug, with a reduction of 59.48% at 4 h in acute inflammation and 83.96% on day 21 in chronic inflammation. Bioassay-guided fractionation using DPPH radical scavenging activity led to isolating and identifying three compounds from EAFMB: oleanolic acid, catechin, and p-hydroxycinnamic acid. All these compounds demonstrated the concentration-dependent inhibition of COX enzymes and the protection of egg albumin from heat-induced denaturation. Catechin exhibited the highest COX inhibition (COX-1 and COX-2 IC_{50} = 9.02 and 50.38 μM, respectively) and anti-denaturation effect (IC_{50} = 27.13 μg/mL) compared to oleanolic acid and p-hydroxycinnamic acid. These isolated compounds are likely responsible for the anti-inflammatory activities of *F. microcarpa* bark.

Keywords: anti-inflammatory activity; *Ficus microcarpa*; catechin; anti-denaturation; COX inhibition

Figure S1a: ^{1}H NMR of FMC-1

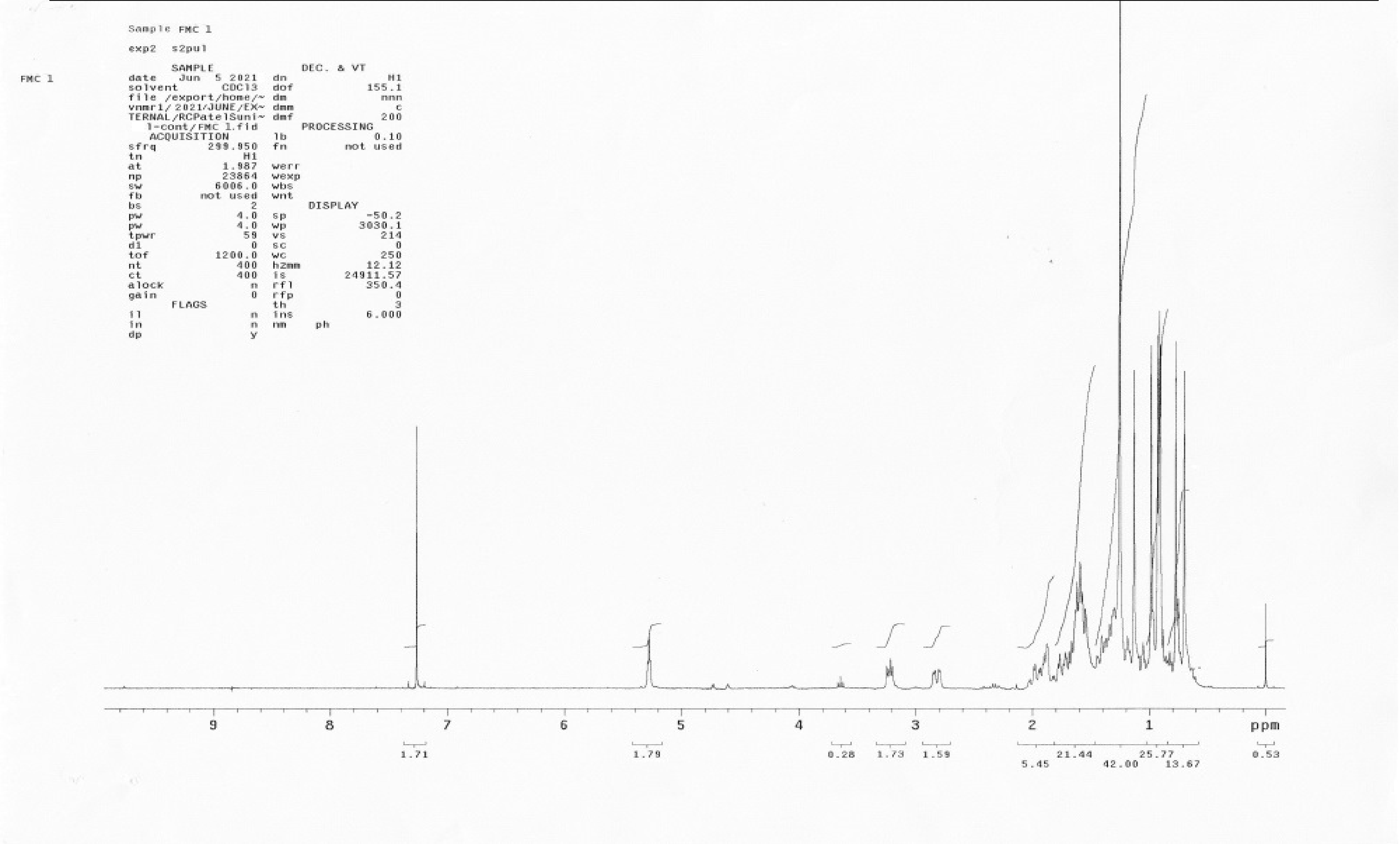

1H NMR of FMC-1 (Resolution)

Sample FMC 1

Data Collected on: quanta-mercuryh1freq
Archive directory: /export/home/vnmr1/vnmrsys/data
Sample directory:
File: FMC 1

Pulse Sequence: s2pul

INDEX	FREQUENCY	PPM	HEIGHT	INDEX	FREQUENCY	PPM	HEIGHT
1	2198.731	7.330	1.7	51	355.946	1.187	12.7
2	2178.570	7.263	63.0	52	350.081	1.167	9.1
3	2175.637	7.253	2.0	53	339.450	1.132	76.6
4	2158.774	7.197	1.6	54	326.254	1.088	7.9
5	1586.547	5.289	6.5	55	315.623	1.052	11.1
6	1583.248	5.278	13.4	56	311.224	1.038	7.0
7	1579.949	5.267	6.8	57	295.461	0.985	82.6
8	1092.767	3.643	2.9	58	290.695	0.969	19.0
9	973.629	3.246	5.4	59	288.863	0.963	16.6
10	968.864	3.230	4.7	60	278.232	0.928	85.1
11	963.365	3.212	7.3	61	273.466	0.912	90.9
12	958.233	3.195	5.3	62	271.267	0.904	83.0
13	854.492	2.849	3.9	63	263.935	0.880	14.2
14	850.459	2.835	4.4	64	256.970	0.857	7.9
15	840.928	2.804	4.7	65	251.838	0.840	6.8
16	836.896	2.790	4.3	66	246.706	0.822	9.0
17	609.251	2.031	1.8	67	241.941	0.807	6.4
18	605.952	2.020	2.2	68	231.677	0.772	83.6
19	596.421	1.988	5.3	69	224.345	0.748	87.3
20	592.389	1.975	5.9	70	215.547	0.719	8.4
21	583.591	1.946	4.6	71	204.550	0.682	2.8
22	579.192	1.931	5.0	72	-0.000	-0.000	20.5
23	572.960	1.910	6.0				
24	569.661	1.899	8.5				
25	562.329	1.875	11.0				
26	559.763	1.866	10.0				
27	546.933	1.823	3.0				
28	542.901	1.810	3.1				
29	533.370	1.778	6.0				
30	528.971	1.764	8.3				
31	519.806	1.733	5.9				
32	515.407	1.718	8.7				
33	512.108	1.707	7.5				
34	505.143	1.684	9.0				
35	498.912	1.663	11.5				
36	489.380	1.632	19.0				
37	485.348	1.618	25.6				
38	477.650	1.592	30.5				
39	471.785	1.573	23.3				
40	464.453	1.548	19.3				
41	434.394	1.448	7.9				
42	431.461	1.438	7.6				
43	422.663	1.409	12.8				
44	411.666	1.372	12.1				
45	405.801	1.353	12.2				
46	401.768	1.339	15.5				
47	395.903	1.320	17.6				
48	390.038	1.300	19.5				
49	375.741	1.253	214.3				
50	359.245	1.198	9.5				

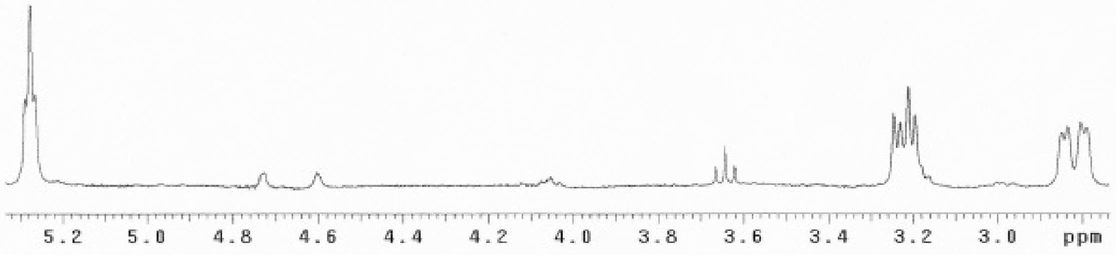

Figure S1b: 13C NMR of FMC-1

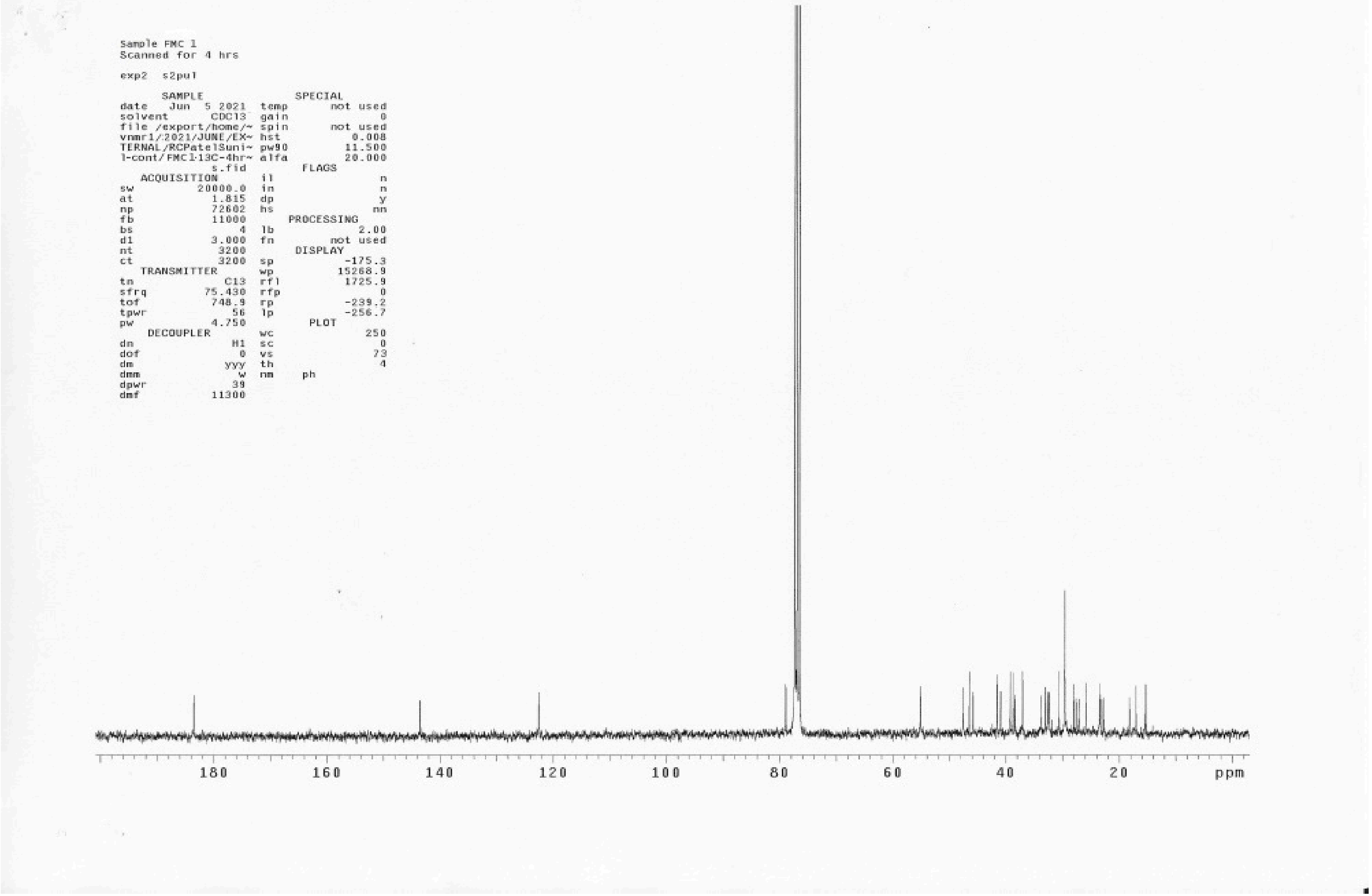

13C NMR of FMC-1 (Resolution

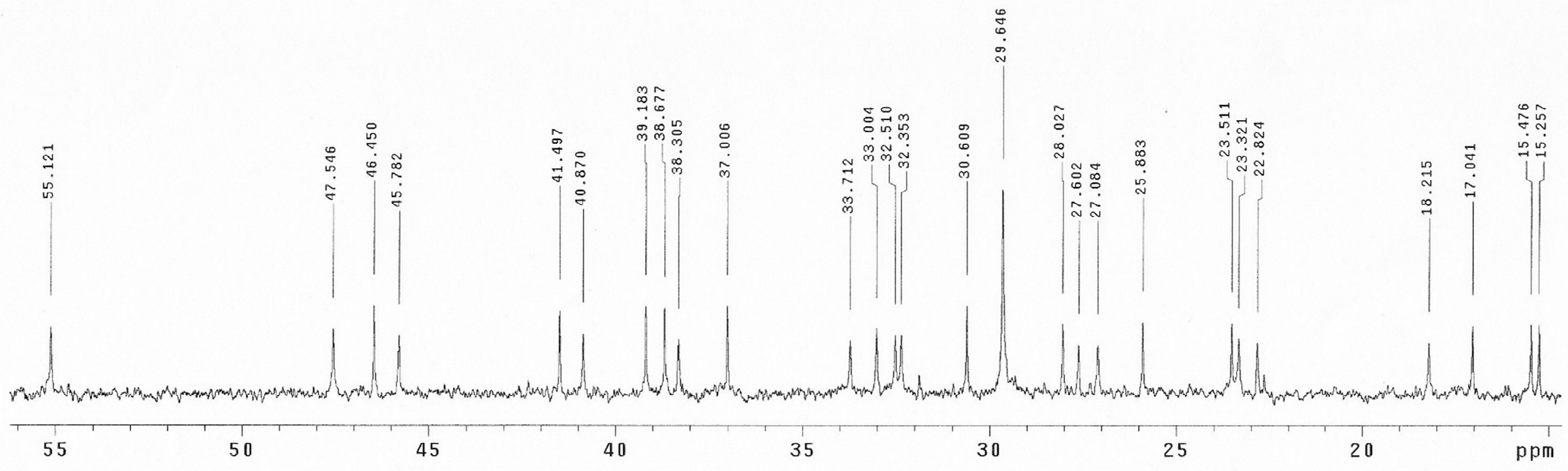

13C NMR of FMC-1 (Resolution

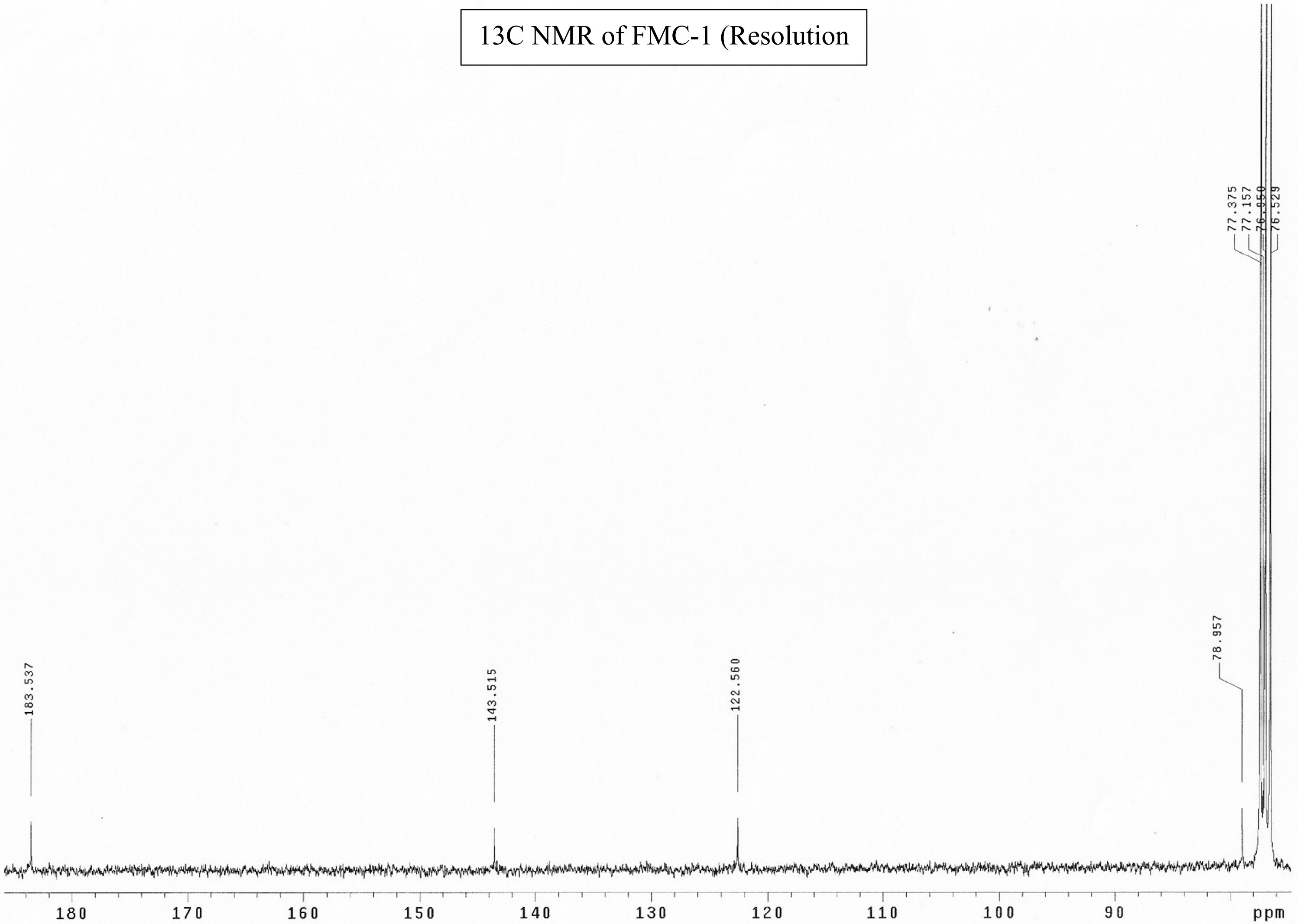

Figure S1c: EI-MS of FMC-1

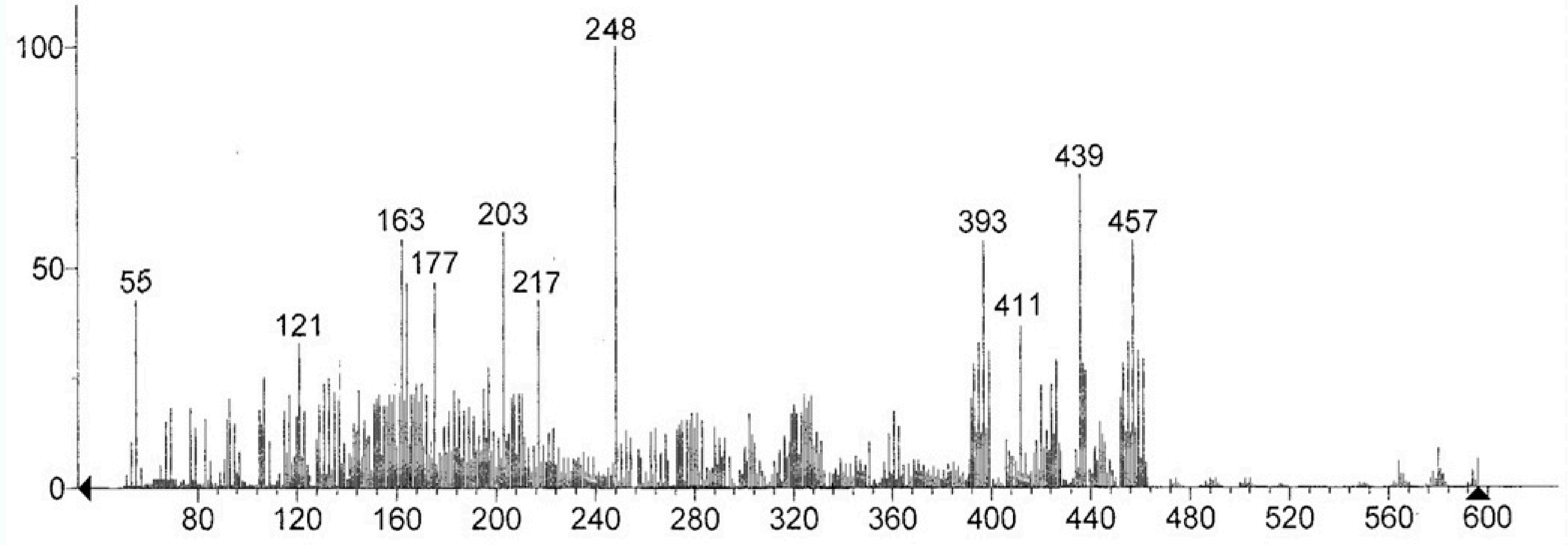

Figure S1d: FT-IR of FMC-1

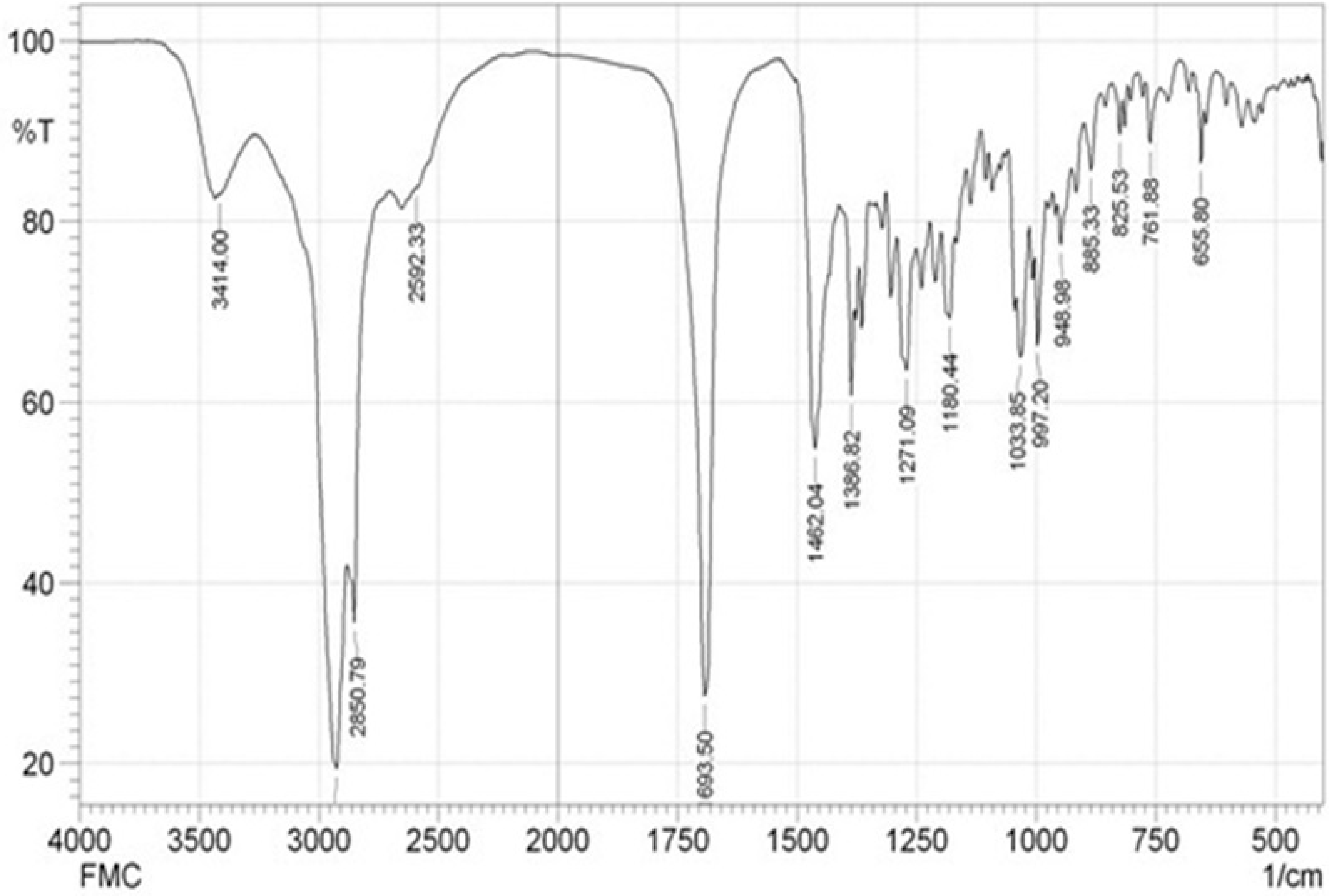

Figure S2a: ^{1}H NMR of FMC-2

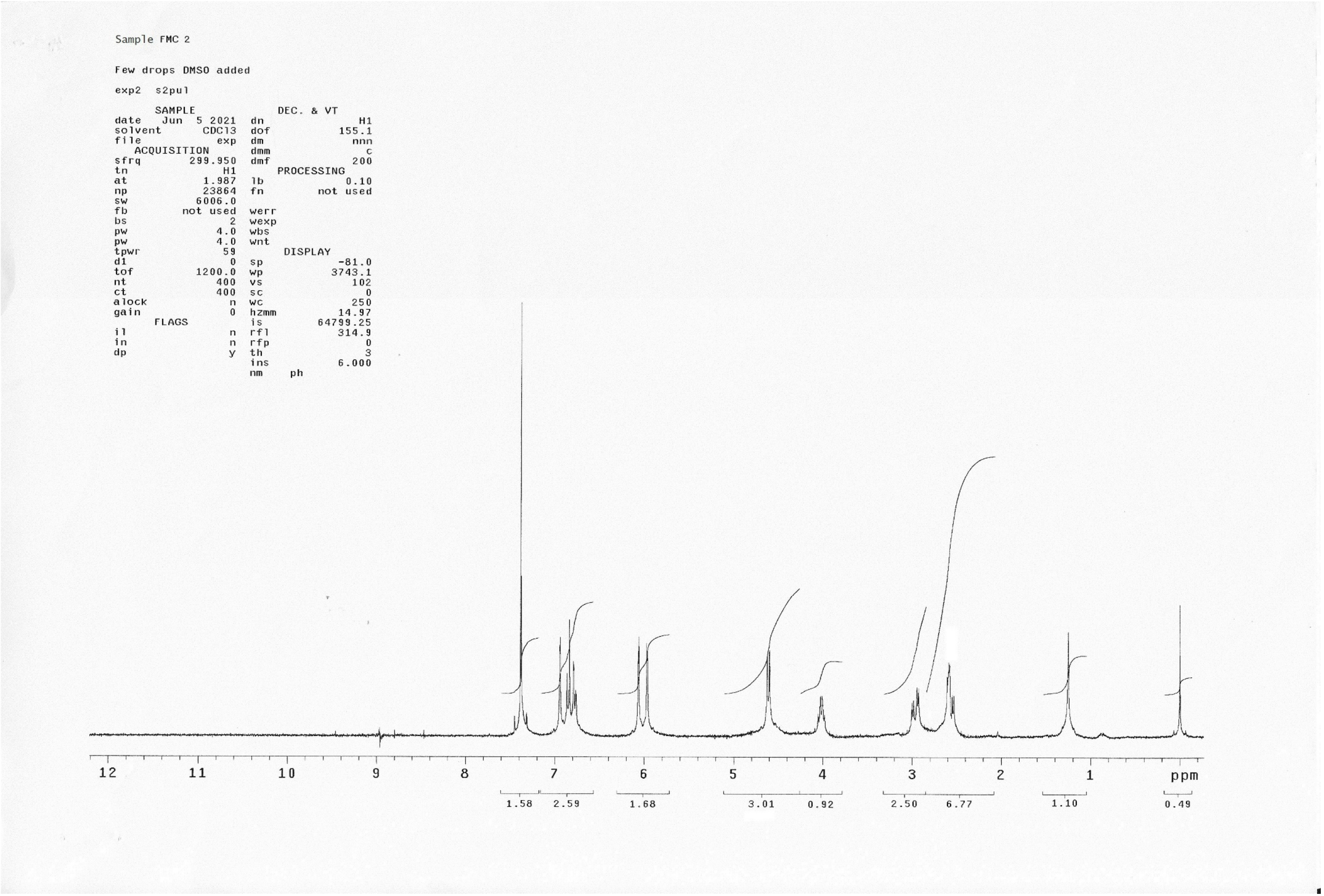

Figure S2b: ^{13}C NMR of FMC-2 (resolution)

Sample FMC 2
Few drops DMSO added

Data Collected on: quanta-mercuryh1freq
Archive directory: /export/home/vnmr1/vnmrsys/data
Sample directory:
File: H1

Pulse Sequence: s2pul

INDEX	FREQUENCY	PPM	HEIGHT
1	2234.657	7.450	4.6
2	2214.495	7.383	102.0
3	2194.333	7.316	5.5
4	2083.993	6.948	21.3
5	2082.527	6.943	23.2
6	2059.432	6.866	14.7
7	2051.368	6.839	27.4
8	2038.171	6.795	17.6
9	2036.338	6.789	16.9
10	2030.106	6.768	10.8
11	1820.057	6.068	20.1
12	1817.858	6.061	23.4
13	1791.098	5.971	21.9
14	1788.898	5.964	20.4
15	1387.129	4.625	18.6
16	1379.431	4.599	20.3
17	1215.204	4.051	5.3
18	1206.773	4.023	9.5
19	1201.641	4.006	9.4
20	1193.576	3.979	5.1
21	900.681	3.003	11.0
22	895.182	2.984	11.6
23	884.552	2.949	14.6
24	879.053	2.931	14.4
25	782.276	2.608	22.5
26	778.977	2.597	23.6
27	777.144	2.591	25.8
28	774.945	2.584	25.4
29	766.514	2.555	17.8
30	758.082	2.527	15.6
31	375.742	1.253	24.7
32	0.000	0.000	31.0

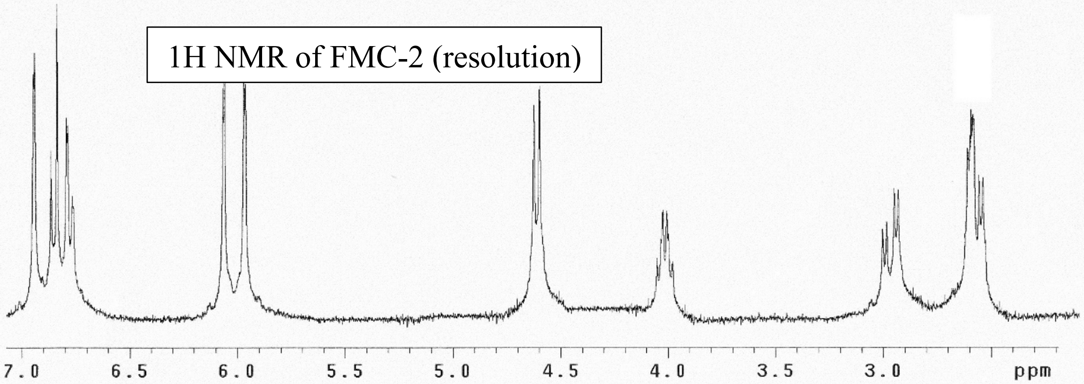

1H NMR of FMC-2 (resolution)

Figure S2c: EI-MS of FMC-2

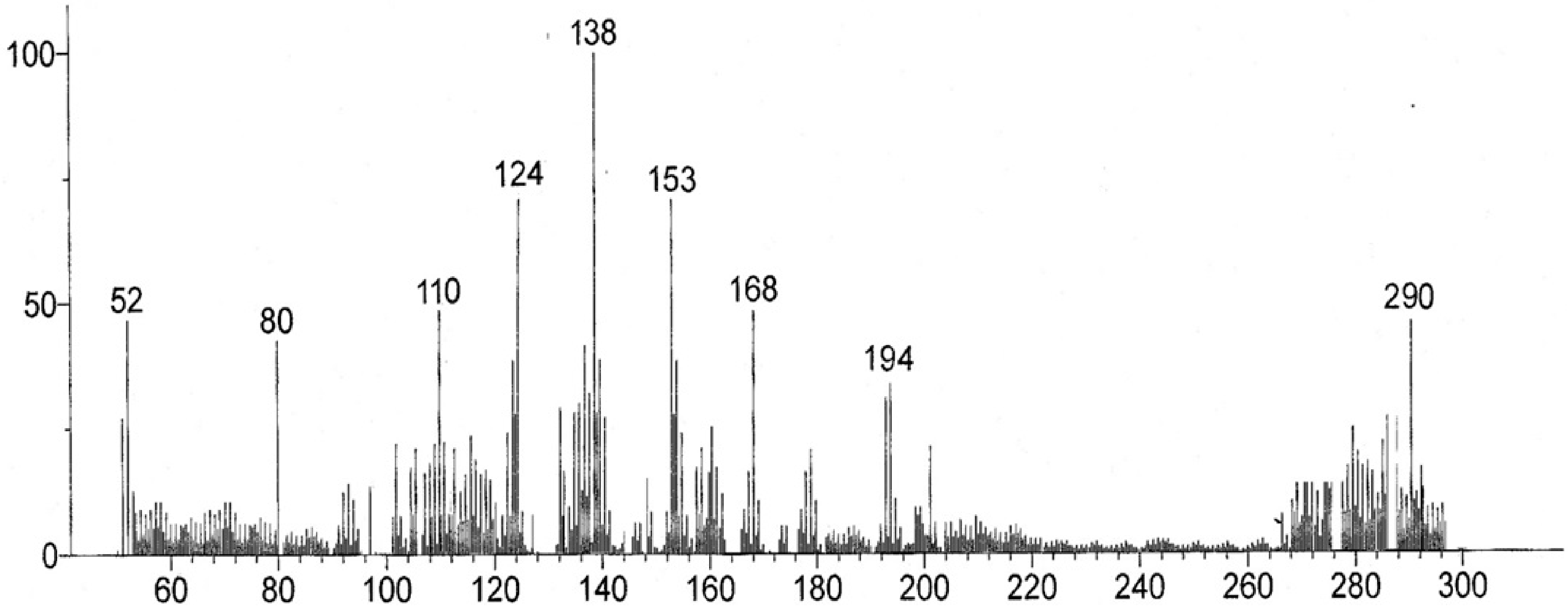

Figure S2d: FT-IR of FMC-2

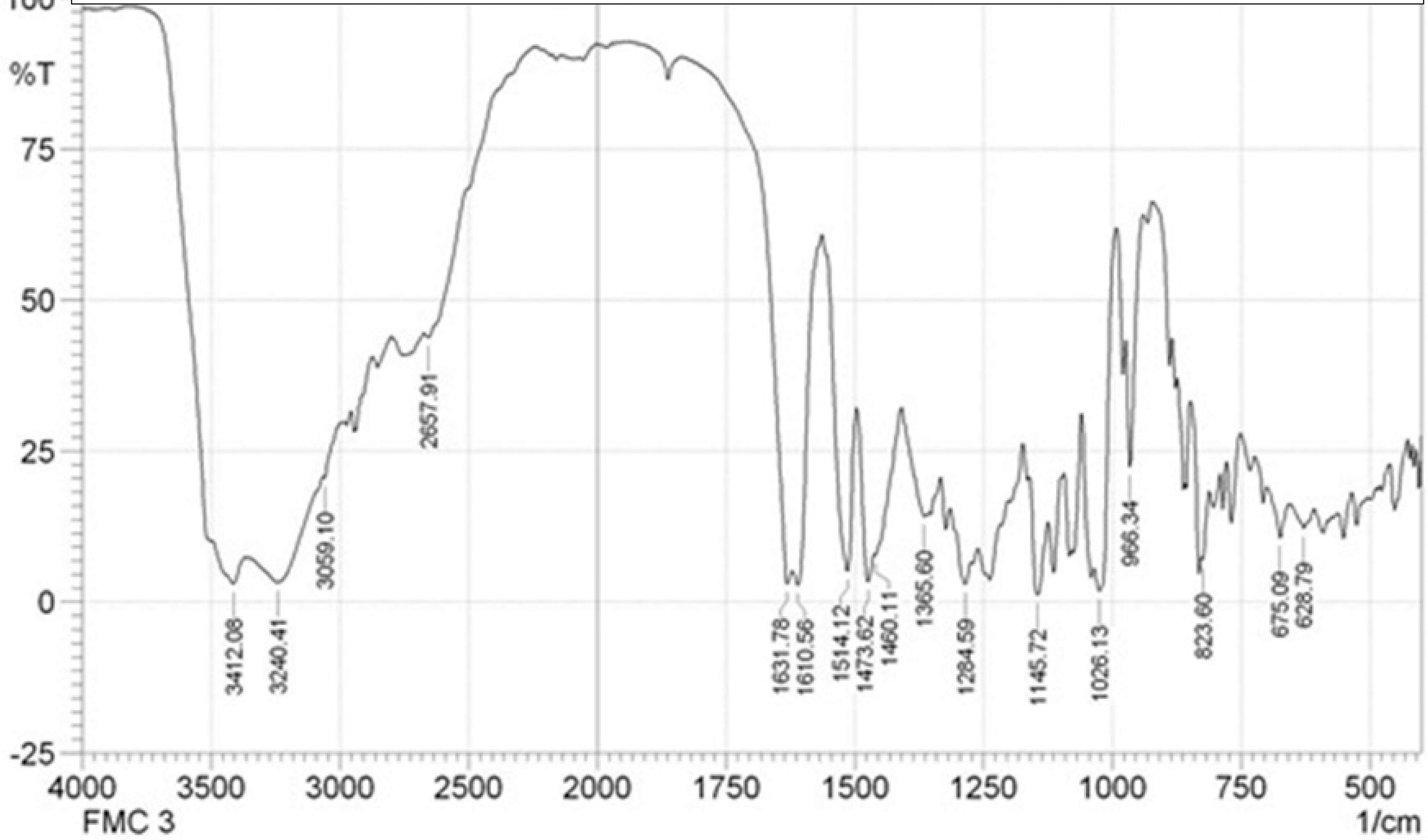

Figure S3a: ^{1}H NMR of FMC-3

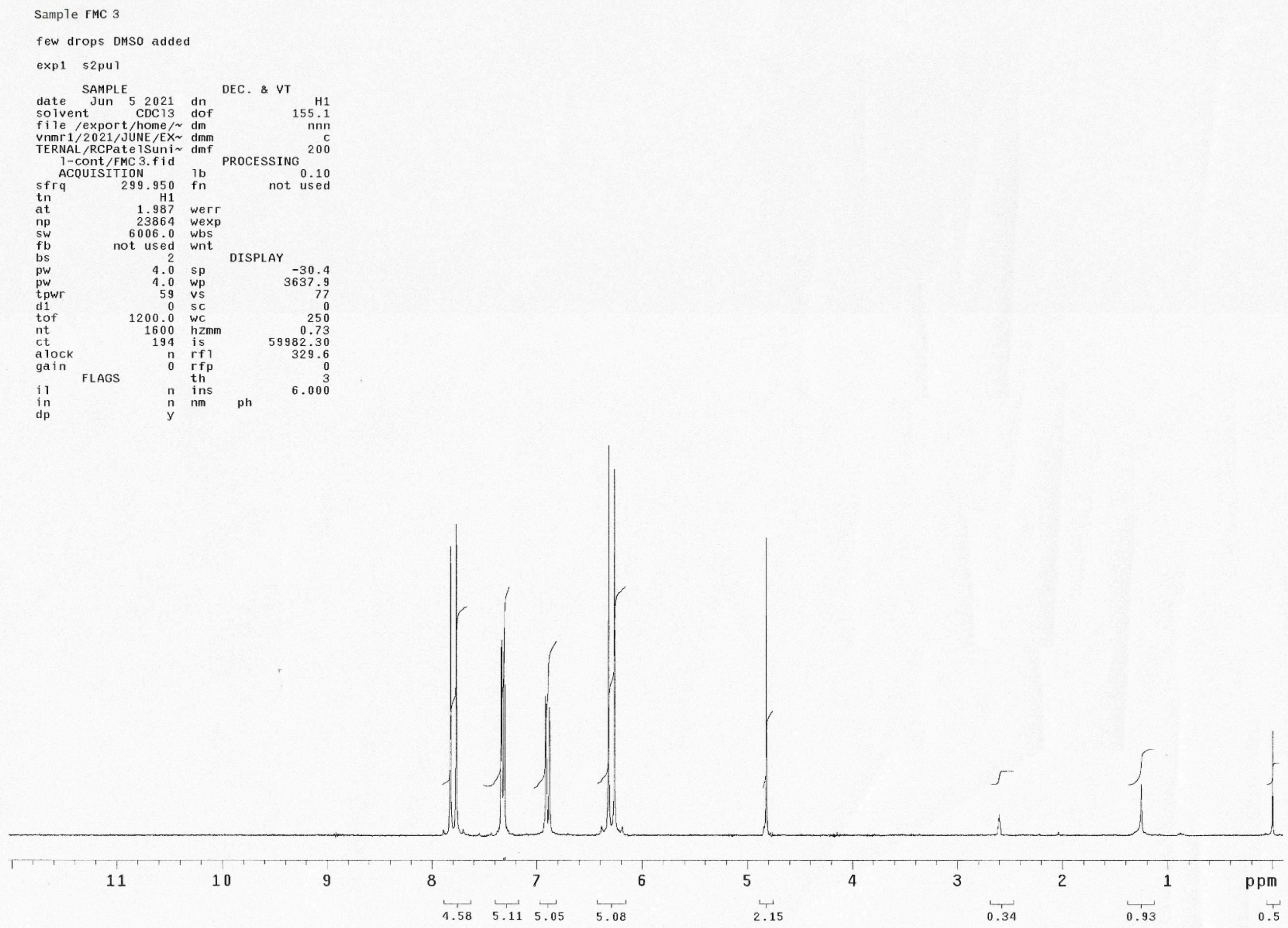

1H NMR of FMC-3 (Resolution)

FMC3
few drops DMSO added

Data Collected on: quanta-mercuryh1freq
Archive directory: /export/home/vnmr1/vnmrsys/data
Sample directory:
File: FMC3

Pulse Sequence: s2pul

INDEX	FREQUENCY	PPM	HEIGHT
1	2392.285	7.596	38.3
2	2235.756	7.544	40.9
3	2146.311	7.341	27.8
4	2144.845	7.314	25.6
5	2076.295	6.919	27.1
6	2068.230	6.858	32.3
7	2049.535	6.446	51.7
8	2042.203	6.402	49.0
9	1962.289	4.782	95.0
10	780.444	2.602	4.9
11	375.375	1.251	10.8
12	-0.733	-0.002	20.6

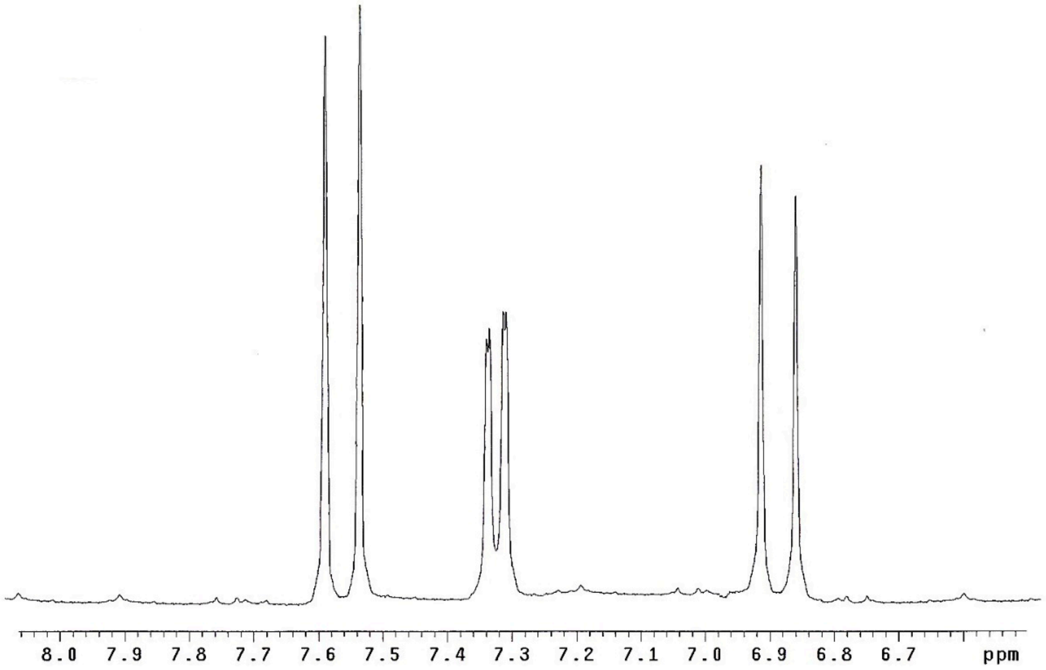

Figure S3b: ^{13}C NMR of FMC-3

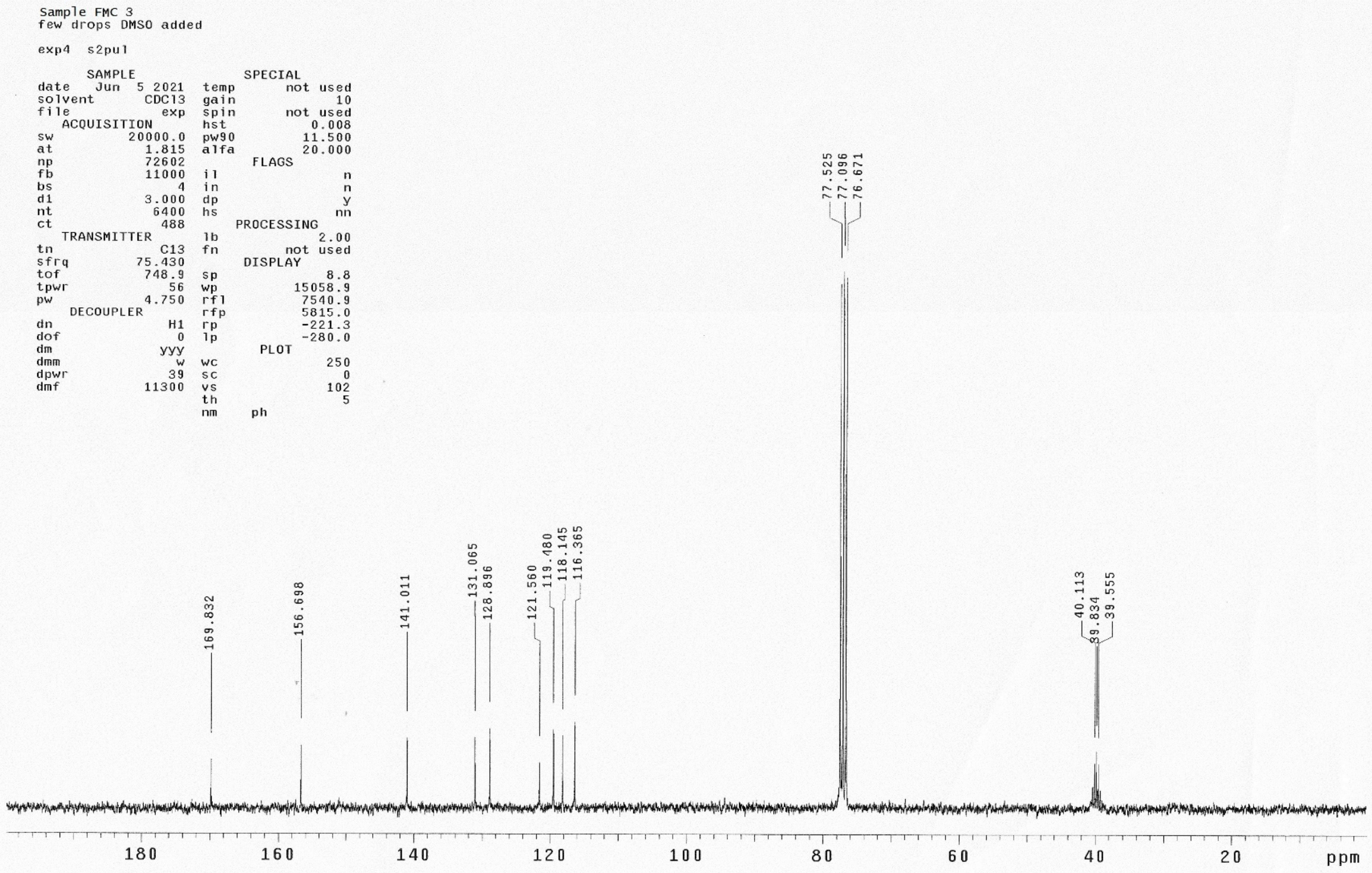

Figure S3d:FT-IR of FMC-3

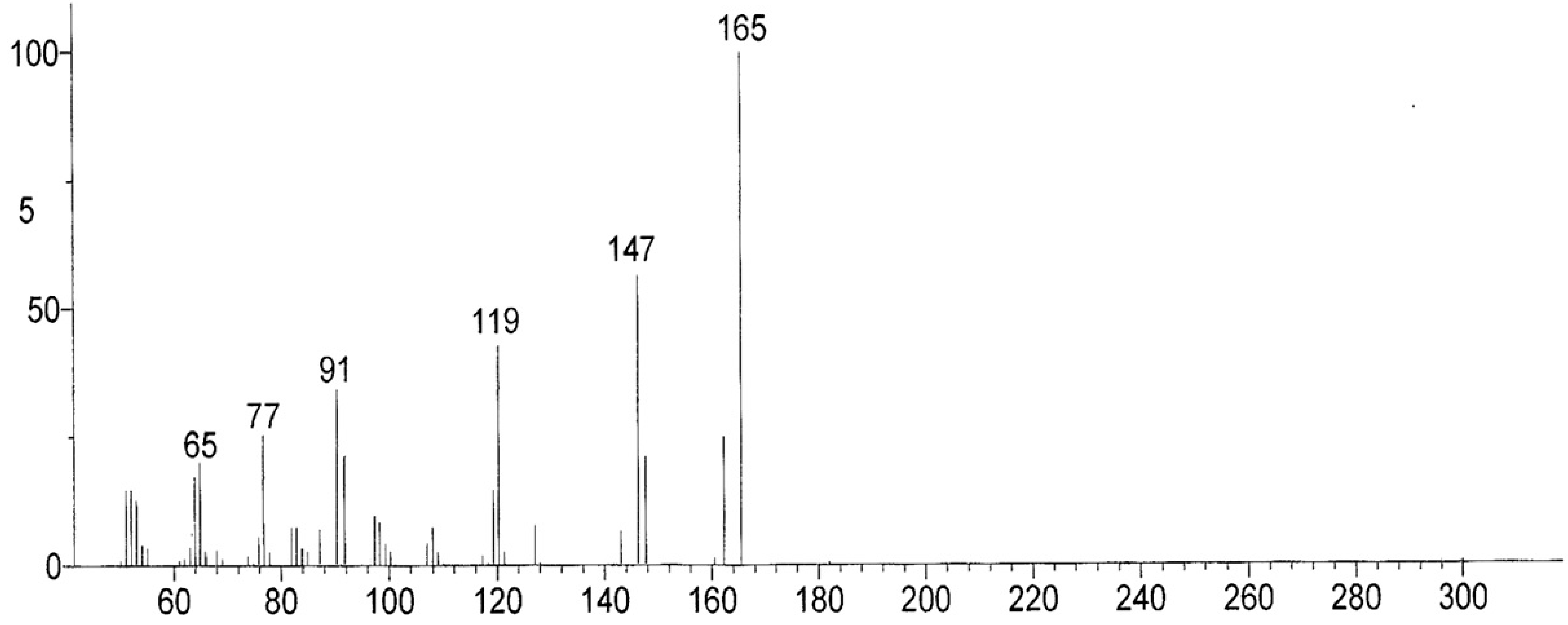

Figure S3c:EI-MS of FMC-3

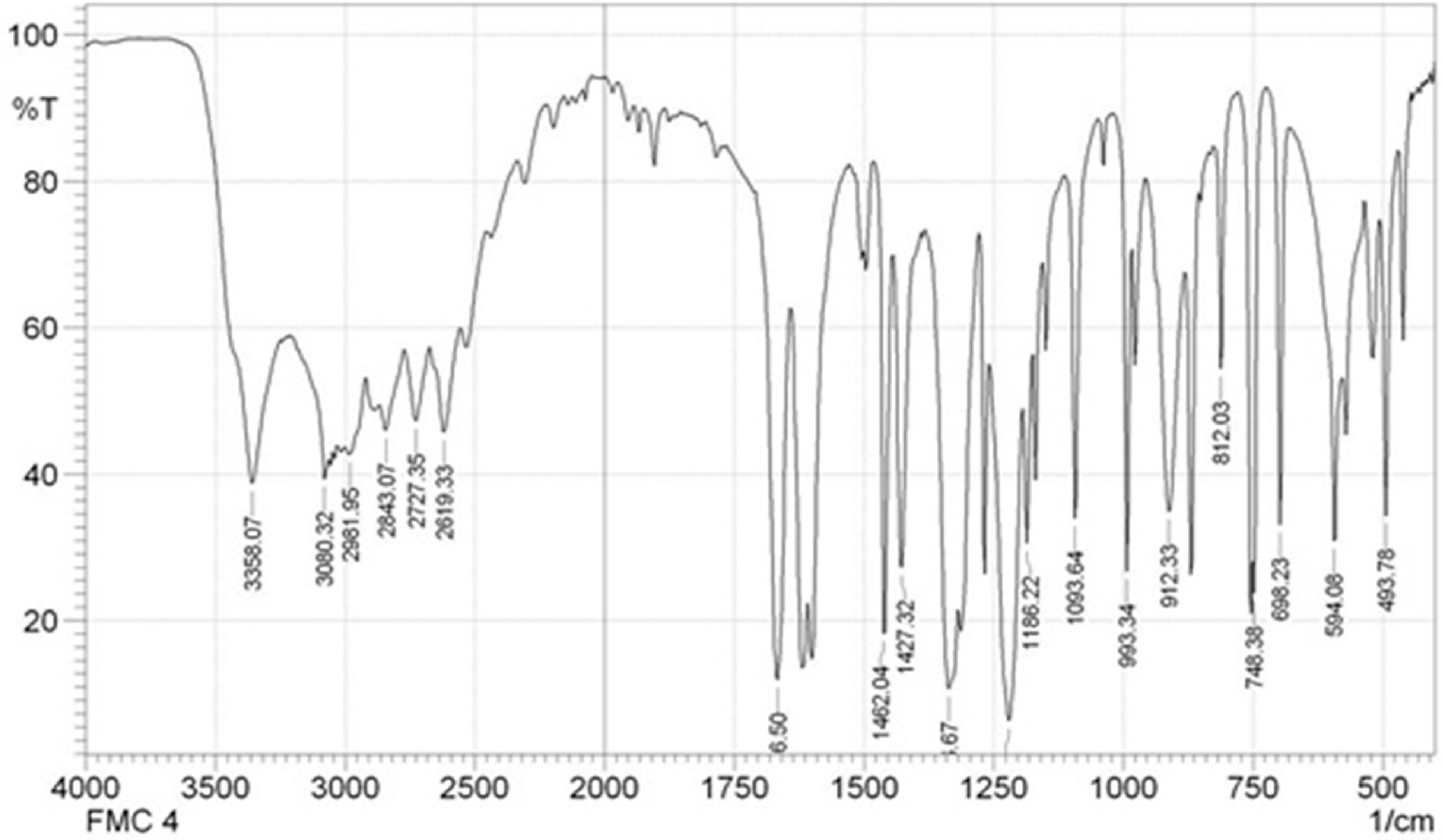

Supplement: Supplementary file 1 [file plants-12-03248-s001.zip › plants-2592577-supplementary.pdf]
